# The Association Between Anthropometric Indices and Type 2 Diabetes Mellitus Among Adults: A Cross‐Sectional Analysis From the Population‐Based Bandare Kong Cohort Study

**DOI:** 10.1002/hsr2.71878

**Published:** 2026-03-02

**Authors:** Seyyed Mohammad Hashemi, Elaheh Salarpour, Parsa Saberian, Arezoo Ghazalgoo, Sara Parandavar, Ehsan Amini‐Salehi, Mohammad‐Hossein Keivanlou, Ladan Hajiabdolrassouli

**Affiliations:** ^1^ Cardiovascular Research Center, Hormozgan University of Medical Sciences Bandar Abbas Iran; ^2^ Endocrinology and Metabolism Research Center, Hormozgan University of Medical Bandar Abbas Iran; ^3^ Student Research Committee, Faculty of Medicine Hormozgan University of Medical Sciences Bandar Abbas Iran; ^4^ School of Medicine, Guilan University of Medical Sciences Rasht Iran

**Keywords:** anthropometric indices, cut‐off values, diabetes, epidemiological research

## Abstract

**Background and Aims:**

Obesity is widely recognized as a significant contributor to the onset of type 2 diabetes mellitus (T2DM). This study aimed to identify the most accurate anthropometric cut‐off points for predicting T2DM in adults aged 35–70 years through a cross‐sectional analysis of the Bandare‐Kong cohort in southern Iran.

**Methods:**

This study analyzed data from the Bandare‐Kong Non‐Communicable Diseases (BKNCD) cohort. Anthropometric indices were analyzed using statistical tests, including *t*‐tests and *χ* ^2^ tests for continuous and categorical variables, respectively. Receiver operating characteristic (ROC) curves and the area under the curve (AUC) were employed to evaluate the diagnostic performance of anthropometric indices in predicting T2DM. A *p*‐value of < 0.05 was considered statistically significant.

**Results:**

A total of 2749 participants were included, evenly distributed between men and women. T2DM was identified in 176 men (6.4%) and 242 women (8.8%). In women, abdominal obesity indicators such as waist‐to‐hip ratio (WHR) (cut‐off: 0.98, AUC = 0.72) and waist‐to‐height ratio (WHtR) (cut‐off: 0.59, AUC = 0.64) demonstrated the strongest associations with T2DM. In men, WHR (cut‐off: 0.94, AUC = 0.65) and WHtR (cut‐off: 0.52, AUC = 0.59) also showed significant associations. When analyzed by age, WHR (cut‐offs: 0.96 for < 50 years and 0.98 for ≥ 50 years) and WHtR (cut‐offs: 0.55 for < 50 years and 0.58 for ≥ 50 years) remained consistently associated with T2DM across age groups. Conversely, body mass index (BMI) showed weaker associations in both genders and age groups.

**Conclusion:**

WHR and WHtR, key indicators of abdominal obesity, demonstrated a strong association with T2DM in both men and women, with WHR showing a particularly stronger relationship in women. These findings highlight the importance of focusing on abdominal obesity measures, especially in women, for better diabetes risk assessment. Future multicenter longitudinal studies are essential to confirm these findings and enhance risk‐stratification approaches.

**Abbreviations**
ADA: American Diabetes Association
AUC: area under the curve
BKNCD: Bandare‐Kong Non‐Communicable Disease
BMI: body mass index
CI: confidence interval
DBP: diastolic blood pressure
FPG: fasting plasma glucose
HC: hip circumference
IDF: International Diabetes Federation
IFG: impaired fasting glucose
MENA: Middle East and North Africa
NHANES: National Health and Nutrition Examination Survey
ORs 95% CIs: odds ratios 95% confidence intervals
PERSIAN: Prospective Epidemiological Research Studies in Iran
ROC: receiver operating characteristic curve
SBP: systolic blood pressure
T2DM: type 2 diabetes mellitus
WC: waist circumference
WHR: waist‐to‐hip ratio
WHtR: waist‐to‐height ratio

## 1 Introduction

The prevalence of diabetes is projected to reach 439 million cases globally by 2030, underscoring its emergence as a significant worldwide epidemic that imposes an escalating burden on individuals and societies [1]. Despite novel and effective treatments [2, 3], diabetes still has a pivotal burden across the world. According to the International Diabetes Federation (IDF), 54.8 million individuals aged 20–79 years in the Middle East and North Africa (MENA) region were living with diabetes as of recent estimates, a figure expected to nearly double to 107.6 million by 2045. Iran, a prominent country within the MENA region, has also been profoundly impacted by this epidemic. In 2018, approximately 5.3 million Iranians were diagnosed with diabetes, with projections suggesting an alarming increase to 9.2 million cases by 2030 [4, 5, 6, 7]. This substantial rise in diabetes prevalence highlights the urgent need to address the disease's burden in Iran, especially given its associated complications [7, 8, 9, 10, 11].

Extensive research has established a robust positive association between T2DM prevalence and excess body weight. Data from the National Health and Nutrition Examination Survey (NHANES) reveal that obesity contributed to 72% of the increase in T2DM cases among both men and women in the US population [12]. Similarly, in Iran, over 30% of the T2DM burden is attributed to excess weight, emphasizing the critical role of obesity in diabetes development [13].

Anthropometric measurements, including BMI, waist circumference (WC), hip circumference (HC), waist‐to‐height ratio (WHtR), and waist‐to‐hip ratio (WHR), are widely used to assess diabetes risk [14, 15, 16]. However, these measures have notable limitations. BMI does not account for fat distribution, and WC may not accurately capture abdominal fat, particularly in populations with varying body shapes [15]. Emerging evidence suggests that WHR and WHtR may serve as more accurate indicators of diabetes risk compared to BMI [17, 18].

WHR, which reflects fat distribution patterns, offers a distinct advantage over BMI and WC by capturing gender‐specific differences in fat storage—men predominantly accumulate fat in the abdominal region, while women tend to store fat in the hips and thighs [19]. Similarly, WHtR is considered superior for diabetes risk prediction as it incorporates both fat distribution around the waist and individual height variability. Studies have demonstrated that WHtR may provide greater predictive reliability than WC [20, 21].

While numerous studies have explored the association between diabetes and obesity indicators, debate persists regarding which anthropometric measurement most accurately reflects body fat distribution and its impact on diabetes risk. Addressing this gap, the present study aims to evaluate the prevalence of diabetes and assess the association between various obesity indices and diabetes. Specifically, this research seeks to determine optimal anthropometric cut‐off points and identify the most reliable indicators of obesity for predicting diabetes in the Bandar Kong population of southern Iran.

## 2 Methods

### 2.1 Study Design and Sampling

This cross‐sectional, population‐based study was conducted as part of the BKNCD research initiative (*N* = 4040), encompassing individuals aged 35–70 years between November 2016 and November 2018. The BKNCD is a subset of the Persian Prospective Epidemiological Research Studies (PERSIAN). Details regarding the study design and methodology have been described comprehensively elsewhere [22]. Participants meeting specific exclusion criteria were omitted from the study. Exclusions included pregnant or breastfeeding individuals, alcohol consumers, and those diagnosed with chronic conditions such as ischemic heart disease, myocardial infarction, stroke, renal failure, thyroid disorders, hepatitis B, hepatitis C, and various cancers. Additionally, individuals using immunosuppressive drugs, those with edema, those adhering to specialized diets, or those with incomplete or insufficient data were excluded. After applying these exclusion criteria, the final analysis included 2749 participants. Among them, 418 individuals were diagnosed with diabetes, comprising 176 males (6.4%) and 242 females (8.8%).


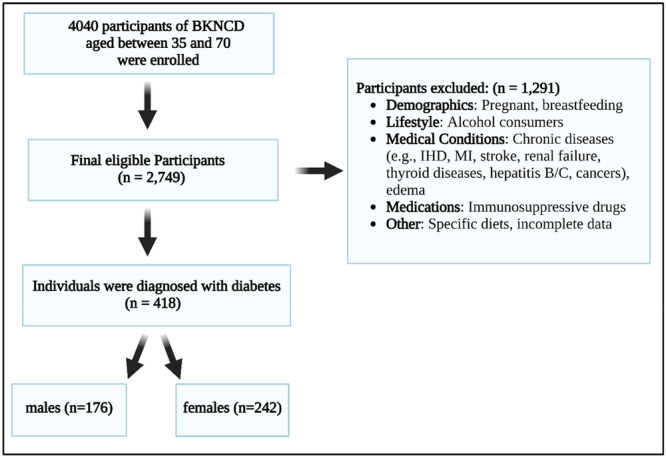


### 2.2 Data Collection

A detailed and standardized questionnaire was employed to collect comprehensive data on participants' demographics, medical history, and medication usage. Trained interviewers conducted all interviews to ensure consistency and accuracy in data collection.

A. Lifestyle and Behavioral Data
  Physical activity was measured through a validated questionnaire that documented participants' daily routines over the previous year. Metabolic equivalent (MET) values assigned to each activity were sourced from an established physical activity compendium. Calculations relied on participants' reported 24‐hour activity patterns, with weekly average MET minutes computed. These minutes reflected various forms of activity, including leisure, occupational, and sports‐related pursuits. These MET minutes captured contributions from activities across leisure, work, and sports contexts [23, 24]. Participants were categorized as smokers or non‐smokers based on their self‐reported answer to whether they had smoked at least 100 cigarettes in their lifetime, with the options being “yes” or “no.” [25].
B. Anthropometric Measurements
  Participants' weight was assessed using a portable scale with a precision of 0.01 kg, ensuring minimal clothing and bare feet for accuracy. Height measurements were taken using a stadiometer to an accuracy of 0.5 cm, with participants standing barefoot, arms relaxed at their sides, and shoulders in a neutral position. BMI was computed by dividing weight in kilograms by height in meters squared. WC was measured to the nearest 0.1 cm with a flexible tape placed horizontally at the midpoint between the lowest rib and the iliac crest. HC was determined at the widest part of the hips while participants maintained a relaxed posture. For accuracy, the mean of the two measurements was recorded. The WHR was calculated as the ratio of WC to HC, and the WHtR was derived by dividing WC by height.
C. Blood Pressure Measurement
  Blood pressure was measured with a mercury sphygmomanometer using an appropriately sized cuff by a trained nurse. Measurements were taken in a seated position following a 5‐min rest period. Two readings, spaced 5 min apart, were averaged for analysis. If there was a discrepancy greater than 10 mmHg in systolic blood pressure (SBP) or 5 mmHg in diastolic blood pressure (DBP), a third measurement was taken, and the average of the two closest values was used. Hypertension was defined as SBP ≥ 140 mmHg or DBP ≥ 90 mmHg [26].
D. Definition of Diabetes
  Diabetes was defined per American Diabetes Association (ADA) criteria as fasting plasma glucose (FPG) ≥ 126 mg/dL, confirmed by repeat testing or glucose‐lowering medications. Impaired fasting glucose (IFG) was defined as FPG between 100 and 126 mg/dL [27]. Participants self‐reporting diabetes or using antidiabetic medications were also included. FPG was measured from blood samples collected after 12 h of fasting using validated diagnostic kits from Pars Azmoun Co. (Tehran, Iran).

### 2.3 Data Analysis

This descriptive, cross‐sectional study used data from the Bandar‐e Kong cohort. The normality of the distribution of continuous variables was assessed using the Kolmogorov–Smirnov test. Categorical variables were reported as counts and percentages, while continuous variables were presented as means ± standard deviations (SD). To compare continuous variables between groups (e.g., male vs. female, age < 50 vs. ≥ 50), a *t*‐test was employed, and the *χ* ^2^ test was used to assess associations between categorical variables.

ROC analysis assessed the diagnostic accuracy of anthropometric indicators and identified optimal cutoff values by gender and age groups (< 50 and ≥ 50 years). These cutoffs categorized anthropometric indices into binary groups (diseased or non‐diseased). The area under the curve (AUC) measured the predictive accuracy for diabetes, with values interpreted as follows: 0.5 (no discrimination), 0.5–0.6 (poor), 0.6–0.7 (weak), 0.7–0.8 (fair), 0.8–0.9 (good), and 0.9–1.0 (excellent). Optimal cutoffs were determined using Youden's index (maximum [sensitivity + specificity − 1]), which identifies the point on the ROC curve that maximizes overall diagnostic accuracy.

Logistic regression models, adjusted for age, further evaluated the predictive ability of anthropometric indices for diabetes, with analyses stratified by gender. Statistical analyses were performed using SPSS (version 25) and MedCalc, the latter specifically for determining optimal cutoff points. A *p*‐value < 0.05 was considered statistically significant.

## 3 Result

This study included a total of 2749 participants, with almost an equal number of males (49.8%) and females (50.2%). The participants' ages ranged from 35 to 70 years old, with an average age of 47.30 years. Out of the total participants, 176 males (6.4%) and 242 females (8.8%) were diabetic. The attributes of the participants are displayed in Table 1.

**Table 1:** Participant characteristics categorized by gender.

Characteristics		Total (*N* = 2749)	Male (*N* = 1370)	Female (*N* = 1379)	*p*‐value
Age	Age (year)	47.30 (9.08)	47.22 (9.15)	47.39 (9.02)	0.613
	< 50	1723 (62.7%)	871 (31.7%)	852 (31.0%)	0.344
	≥ 50	1026 (37.3%)	499 (18.2%)	527 (19.2%)	
Residence	Urban	2322 (84.5%)	1179 (42.9%)	1143 (41.6%)	0.024
	Rural	427 (15.5%)	191 (6.9%)	236 (8.6%)	
Socio‐economic status	Low	1071 (39.1%)	451 (16.5%)	620 (22.6%)	< 0.001
	Moderate	528 (19.3%)	279 (10.2%)	249 (9.1%)	
	High	1142 (41.6%)	632 (23.1%)	510 (18.5%)	
Physical activity score (METs/day)	Low (24–36.5)	640 (23.6%)	333 (12.3%)	307 (11.3%)	< 0.001
	Moderate (36.6–44.9)	1638 (60.5%)	728 (26.9%)	910 (33.6%)	
	Vigorous (> = 45)	431 (15.9%)	288 (10.6%)	143 (5.3%)	
Cigarette smoking	Yes	438 (15.9%)	434 (15.8%)	4 (1.0%)	< 0.001
	No	2311 (84.1%)	936 (34.0%)	1375 (50.0%)	
Hypertension	Yes	747 (27.2%)	376 (13.7%)	371 (13.5%)	0.764
	No	2002 (72.8%)	994 (36.2%)	1008 (36.7%)	
Diabetes	Yes	418 (15.2%)	176 (6.4%)	242 (8.8%)	0.001
	No	2331 (84.8%)	1194 (43.4%)	1137 (41.4%)	
Weight(Kg)		70.56 (14.09)	74.46 (14.16)	66.68 (12.90)	< 0.001
Height(m)		1.63 (0.09)	1.70 (0.06)	1.56 (0.05)	< 0.001
WC (cm)		92.44 (11.47)	89.86 (11.00)	95.01 (11.36)	< 0.001
HC (cm)		99.25 (8.94)	97.59 (8.11)	100.91 (9.42)	< 0.001
BMI(kg/m^2^)		26.39 (4.73)	25.60 (4.36)	27.18 (4.96)	< 0.001
WHR		0.93 (0.66)	0.92 (0.06)	0.94 (0.07)	< 0.001
WHtR		0.57 (0.08)	0.53 (0.06)	0.61 (0.07)	< 0.001
SBP (mmHg)		117.63 (16.78)	119.59 (15.94)	115.68 (17.36)	< 0.001
DBP (mmHg)		76.45 (10.23)	78.01 (9.73)	74.91 (10.48)	< 0.001

*Note:* Categorical variables are represented using numerical values and percentages (%), whereas continuous variables are represented using the mean and standard deviation (SD). The independent‐samples *t*‐test is used for comparing continuous data, while the *χ* ^2^ test is used for comparing categorical variables.

Abbreviations: BMI, body mass index; DBP, diastolic blood pressure; HC, hip circumference; SBP, systolic blood pressure; WC, waist circumference; WHR, waist‐to‐hip ratio; WHtR, waist‐to‐height ratio.

**Figure 1:**
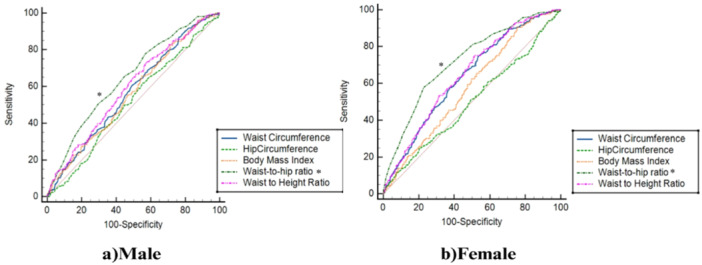
Receiver Operating Characteristic (ROC) curves illustrating the predictive performance of anthropometric indices (WC, HC, BMI, WHR, WHtR) for type 2 diabetes mellitus (T2DM) in 2749 adults aged 35–70 years from the Bandare‐Kong cohort, stratified by gender. WHR (AUC = 0.72 in women, 0.65 in men) and WHtR (AUC = 0.64 in women, 0.59 in men) showed superior performance compared to BMI.

**Figure 2:**
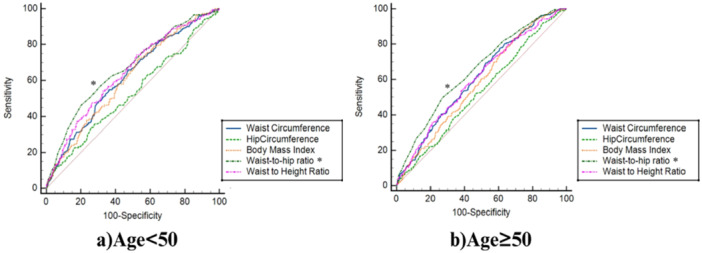
ROC curves showing the association between anthropometric indices (WC, HC, BMI, WHR, WHtR) and T2DM in 2749 adults aged 35–70 years from the Bandare‐Kong cohort, stratified by age (< 50 and ≥ 50 years). WHR (AUC = 0.67 for < 50 years, 0.65 for ≥ 50 years) and WHtR (AUC = 0.64 for < 50 years, 0.60 for ≥ 50 years) outperformed other indices.

The ROC analysis based on gender and age, as demonstrated in Figure 1, indicates that WHR and WHtR are more efficient measures than other indices for assessing both female and male individuals. Furthermore, it must be noted that, in females, aside from WHR and WHtR, WC also exhibited acceptable performance compared to other indices. Moreover, as depicted in Figure 2, anthropometric indices like WHR, WHtR, and WC displayed relatively similar and adequate predictive ability for diagnosed diabetes in individuals below the age of 50, and regarding those aged 50 or above.

Table 2 provides the AUC and optimal cut‐off points for various anthropometric measures, highlighting their association with T2DM across genders. Among men, the WHR showed the strongest association with T2DM, with an AUC of 0.65 (95% CI: 0.62–0.67) and a cut‐off value of 0.94. Similarly, for women, WHR demonstrated an even stronger relationship, with an AUC of 0.72 (95% CI: 0.69–0.74) and a cut‐off value of 0.98. While WHtR also showed a reasonable level of association in both men (AUC: 0.59) and women (AUC: 0.64), BMI exhibited weaker associations, particularly in men (AUC: 0.56) and to a slightly lesser extent in women (AUC: 0.58). These results indicate that WHR, followed by WHtR, is a more reliable measure for assessing the association with T2DM compared to BMI.

**Table 2:** The AUC and optimal cut‐off points demonstrate the relationship between body measurement metrics and T2DM across genders.

Measures	Male			Female		
	AUC (99% CI)	*p* value	Cut‐off‐value	AUC (99% CI)	*p* value	Cut‐off‐value
WC (cm)	0.57 (0.54–0.59)	0.002	89.6	0.64 (0.61–0.66)	< 0.001	92.7
HC (cm)	0.52 (0.48–0.54)	0.476	98.5	0.50 (0.47–0.53)	0.976	92.8
BMI (kg/m^2^)	0.56 (0.53–0.58)	0.006	22.7	0.58 (0.55–0.60)	< 0.001	23.33
WHR	0.65 (0.62–0.67)	< 0.001	0.94	0.72 (0.69–0.74)	< 0.001	0.98
WHtR	0.59 (0.56–0.62)	< 0.001	0.52	0.64 (0.62–0.67)	< 0.001	0.59

Table 3 explores the AUC and optimal cut‐off points for body measurement metrics across different age groups (< 50 and ≥ 50 years). Among participants younger than 50 years, WHR displayed the highest level of association with T2DM, with an AUC of 0.67 (95% CI: 0.64–0.69) and a cut‐off value of 0.96, surpassing WHtR (AUC: 0.64) and BMI (AUC: 0.61). In participants aged 50 years and above, WHR remained the most strongly associated measure, with an AUC of 0.65 (95% CI: 0.62–0.68) and a cut‐off value of 0.98. WHtR showed a moderate association (AUC: 0.60), whereas BMI again exhibited weaker performance (AUC: 0.58). These findings reinforce WHR as the reliable indicator of the relationship between anthropometric indices and T2DM, followed by WHtR, while BMI was less effective in capturing this association.

**Table 3:** The AUC and optimal cut‐off points illustrate the relationship between body measurement metrics and T2DM across different age groups.

Measures	Age < 50			Age ≥ 50		
	AUC (99% CI)	*p* value	Cut‐off‐value	AUC (99% CI)	*p* value	Cut‐off‐value
WC (cm)	0.62 (0.59–0.64)	< 0.001	95.1	0.61 (0.58–0.64)	< 0.001	90.9
HC (cm)	0.53 (0.51–0.55)	0.231	104.8	0.53 (0.50–0.56)	0.122	90.1
BMI (kg/m^2^)	0.61 (0.58–0.63)	< 0.001	25.60	0.58 (0.55–0.61)	< 0.001	23.29
WHR	0.67 (0.64–0.69)	< 0.001	0.96	0.65 (0.62–0.68)	< 0.001	0.98
WHtR	0.64 (0.62–0.66)	< 0.001	0.55	0.60 (0.57–0.63)	< 0.001	0.58

Based on the results of the multivariable logistic regression analysis, the strength of association between anthropometric indices and T2DM varied by gender. Among men, BMI (OR: 1.91, 95% CI: 1.25–2.92, *p* = 0.003) showed the strongest association, closely followed by WHR (OR: 1.89, 95% CI: 1.34–2.65, *p* < 0.001), WHtR (OR: 1.67, 95% CI: 1.18–2.38, *p* = 0.004), and WC (OR: 1.65, 95% CI: 1.17–2.33, *p* = 0.004). HC showed no significant association. In women, WHR demonstrated the strongest association (OR: 3.76, 95% CI: 2.77–5.10, *p* < 0.001), followed by BMI (OR: 3.19, 95% CI: 1.99–5.10, *p* < 0.001), WC (OR: 2.39, 95% CI: 1.73–3.30, *p* < 0.001), and WHtR (OR: 2.30, 95% CI: 1.66–3.19, *p* < 0.001), with HC again showing no significant association (Table 4). These findings highlight the varying strengths of association between different anthropometric indices and T2DM across genders, with WHR showing a particularly strong association in women.

**Table 4:** Analysis of the association between anthropometric variables, categorized by specified cut‐off values, and the existence of T2DM using multivariable logistic regression.

Measures	Male			Female		
	Odds ratio	95% CI	*p*‐value	Odds ratio	95% CI	*p*‐value
WC (cm)	1.65	1.17–2.33	0.004	2.39	1.73–3.30	< 0.001
HC (cm)	1.15	0.81–1.65	0.420	1.23	0.87–1.75	0.240
BMI (kg/m^2^)	1.91	1.25–2.92	0.003	3.19	1.99–5.10	< 0.001
WHR	1.89	1.34–2.65	< 0.001	3.76	2.77–5.10	< 0.001
WHtR	1.67	1.18–2.38	0.004	2.30	1.66–3.19	< 0.001

*Note:* For males, references were Waist ≤ 89.6 cm, Hip ≤ 98.5 cm, BMI ≤ 22.7 kg/m2, WHR ≤ 0.94 cm, and WHtR < 0.52 cm. For females, reference cut‐off values included WC < 92.7 cm, HC ≤ 92.8 cm, BMI ≤ 23.3 kg/m2, WHR ≤ 0.98 cm, and WHtR ≤ 0.59 cm. Adjusted for age, socioeconomic level, smoking, and physical activity.

Abbreviations: BMI, body mass index; HC, hip circumference; WC, waist circumference; WHR, waist‐to‐hip ratio; WHtR, waist‐to‐height ratio.

## 4 Discussion

The aim of this study was to identify precise anthropometric indicators linked to type 2 diabetes (T2DM) in adults from southern Iran. The results revealed that measures of abdominal obesity, particularly WHR and WHtR, exhibited a stronger association with T2DM than general indicators such as BMI in both genders. Notably, the WHR threshold for women (0.98) was higher than that for men (0.94), reflecting gender‐based differences in fat distribution. Similarly, the WHtR cut‐off was higher in women (0.59) compared to men (0.52), underscoring the significance of central obesity in its relationship with diabetes. In contrast, the BMI cut‐off values for diabetes were nearly the same for both males (22.7 kg/m²) and females (23.33 kg/m²). These findings emphasize the crucial role of abdominal obesity metrics, particularly WHR and WHtR, in predicting diabetes risk and highlight the necessity of gender‐specific cut‐off points for this population.

WHR and WHtR offer valuable insights into abdominal obesity, which is a crucial factor in the development of T2DM. A study by Khader et al. in Jordan highlighted that WHtR, WHR, and WC outperformed other anthropometric indices, including BMI, in their association with diabetes for both sexes. They identified WHtR as the most effective measure, with optimal cut‐off values of 0.6 for women and 0.57 for men [28]. Similarly, Woldegebriel et al. demonstrated the superiority of abdominal obesity indices over BMI in both genders, reporting that WHtR (cut‐off: 0.52) in men and WC (cut‐off: 83.5 cm) in women had the strongest associations with diabetes [29]. Consistent with these findings, Sadeghi et al. in Iran also found WHR and WHtR to be superior to BMI. Among men, the optimal cut‐off points for WHR and WHtR were 0.91 and 0.52, respectively, while for women, the values were 0.80 and 0.55 [30]. Furthermore, a subsequent study by Arslan et al. highlighted the predictive value of WHR and WHtR for cardiovascular risk in patients with T2DM [31]. Recent work by Arslan et al. demonstrated a significant association between dietary intake, serum advanced glycation end products, and diabetic complications, underscoring the metabolic consequences of poor glycemic control [32]. These findings reinforce the clinical utility of central obesity measures in both metabolic and cardiovascular risk assessment.

A study conducted in Yazd City, Iran, demonstrated that WHtR had superior discriminatory ability for diabetes risk in both men (AUC = 0.692) and women (AUC = 0.708) compared to BMI, which showed lower discriminatory power (AUC = 0.60 for men and AUC = 0.63 for women). The WHtR cut‐off threshold was higher in women (0.605) than in men (0.56), aligning with the findings of our study. In contrast, for WHR, men exhibited a higher threshold (0.939) compared to women (0.892) [33]. Additionally, a cohort study by Zafari et al. in Tehran highlighted WHtR as the most effective index for distinguishing diabetes risk in both sexes. The optimal cut‐off points for BMI, WC, WHtR, WHR, and HC were reported as 25.56 kg/m², 89 cm, 0.52, 0.91, and 96 cm for men, and 27.12 kg/m², 87 cm, 0.56, 0.83, and 103 cm for women, respectively [34].

Although the cut‐off points in our study slightly differ from these values, these discrepancies may be attributed to population‐specific factors, such as genetic, lifestyle, and environmental differences. For instance, research conducted in Germany also found that abdominal obesity indices like WC and WHtR had stronger associations with T2DM onset compared to BMI and weight, further reinforcing WHtR as the most reliable indicator of diabetes risk [35].

WHR and WHtR are valuable anthropometric measures that assess fat distribution, particularly central adiposity, which plays a critical role in the development of T2DM. The primary mechanism linking central adiposity to T2DM lies in the effects of visceral fat, which promotes insulin resistance by releasing free fatty acids, inflammatory cytokines, and other bioactive molecules. Elevated WHR, indicative of increased visceral fat, is strongly associated with a heightened risk of diabetes [36, 37, 38].

WHtR offers additional advantages as it accounts for variations in body size and shape, enhancing its applicability across diverse populations. Unlike BMI, which reflects overall body weight without distinguishing fat distribution, WHtR focuses on abdominal fat, which is more strongly correlated with metabolic and cardiovascular risks. This specificity makes WHtR a superior measure for identifying individuals at risk of diabetes and related complications [39].

While BMI is a commonly used metric in clinical practice due to its simplicity, it has notable limitations. Specifically, BMI does not distinguish between individuals with high muscle mass and those with excess fat, potentially leading to misclassification of obesity‐related health risks [40]. Despite these drawbacks, BMI continues to be a relevant marker for diabetes risk. For example, in Yang's study, BMI emerged as the most significant anthropometric index associated with diabetes in elderly men (AUC = 0.655) and women (AUC = 0.635), with optimal cut‐off points of 25.78 kg/m² for men and 24.86 kg/m² for women in the elderly Chinese population [41]. Similarly, findings from the Health Professionals Follow‐Up Study revealed that BMI and WC had comparable associations with T2DM risk in men aged 40–75 years. In contrast, WHR demonstrated weaker associations, highlighting differences in the utility of various anthropometric measures across populations and age groups [42].

In our study, after adjusting for confounding factors, we found that in men, both BMI, as an indicator of general obesity (OR: 1.91), and WHR (OR: 1.89) were associated with nearly double the risk of diabetes, with BMI showing a slightly stronger association. In women, however, WHR emerged as the most significant factor, being linked to a fourfold higher risk of developing diabetes (OR: 3.76), indicating its dominant role in this group. BMI was also notably associated with diabetes risk in women, with a threefold increase (OR: 3.19), making it the second most significant index in this group. These findings underscore the gender‐specific variations in the relationship between anthropometric indices and diabetes risk, with a notably stronger association observed for WHR in women.

One possible reason why WHR serves as a significant indicator in females is the gender‐specific variations in visceral fat accumulation and the distribution of fat across different regions [43]. This may also be influenced by the characteristics of our study population, which predominantly consisted of middle‐aged and elderly individuals. In postmenopausal women, hormonal changes during menopause result in a significant redistribution of body fat, leading to increased abdominal fat accumulation. This change strengthens the relationship between central obesity and metabolic risks, including diabetes [44]. As a result, the link between visceral adipose tissue and diabetes risk factors is typically more pronounced in females [45, 46]. These results align with cross‐ethnic studies and case‐control research, which consistently identify central obesity as a major risk factor for diabetes in women.

Additionally, researchers such as Noel et al. have questioned BMI's ability to accurately reflect obesity and effectively differentiate between obese and non‐obese individuals [47]. Indeed, BMI fails to distinguish between lean body mass and fat mass, and it does not adequately assess body fat distribution—an essential determinant of metabolic risk [39]. These limitations highlight the need to incorporate measures like WHR for a more accurate assessment of diabetes risk, especially in women [48].

In the Zabetian research, it was observed that both general obesity (BMI = 30 kg/m²) and elevated WHR serve as key predictors of type 2 diabetes in the Iranian population younger than 60. However, for individuals aged 60 and above, increased WC emerged as the sole predictor of diabetes. Therefore, it is important to factor in age when employing anthropometric measurements to assess the risk of type 2 diabetes [49, 50] In our study, when examining the association between age and diabetes in individuals under 50, the anthropometric indices WHR and WHtR showed stronger relationships with diabetes compared to other measures, with cut‐off values of 0.96 for WHR and 0.55 for WHtR. Additionally, in participants aged over 50, WHR remained highly associated with diabetes, with a cut‐off value of 0.98.

Furthermore, Snijder et al. have shown that WHR is a predictor of diabetes onset in the young age group, but its predictive power decreases or disappears in the older age group [51]. While most studies have demonstrated WC as the best anthropometric predictor of diabetes onset in young individuals, and have suggested that WC reflects intra‐abdominal fat accumulation better than WHR [20]. However, it is important to note that an increase in WHR indicates both relative abdominal fat accumulation or an increase in WC and relative gluteal muscle reduction or a decrease in HC [52]. Additionally, it is noteworthy that the lack of association between HC and T2DM in our study may be due to its reflection of subcutaneous fat, which is less metabolically active than visceral fat [53].

The observed variations in findings can be attributed to several factors. Primarily, the effectiveness of each anthropometric measurement is specific to different populations and differs across racial groups [54, 55]. For example, Lear's research indicates that racial variations influence the association between WC and metabolic risk factors [56]. Secondly, another justifiable reason for this difference is related to variations in measurement methods [38, 57].

The results of this study are important for developing countries, particularly countries such as Iran, where urbanization has brought about lifestyle changes that may contribute to alterations in nutrition and demographics. As a result, there has been an unanticipated rise in the prevalence of non‐communicable diseases (NCDs) like diabetes mellitus, which has imposed a significant strain on the country [58]. Therefore, the results of our study can be utilized to inform the development of targeted programs aimed at preventing NCDs, specifically diabetes.

### 4.1 Strengths and Limitations

This study's key strength includes its large, population‐based sample, which enhances the generalizability of the results to middle‐aged and elderly individuals in southern Iran. The use of standardized protocols for anthropometric measurements, such as WC and WHR, ensures reliable and consistent data collection. By accounting for confounding factors like age, physical activity, and socioeconomic status, the analysis provides a robust assessment of the relationship between anthropometric indices and T2DM. Moreover, the study's evaluation of multiple indices—WHR, WHtR, and BMI—offers a comprehensive comparison of their relevance in diabetes risk assessment across genders and age groups. The inclusion of gender‐ and age‐specific cut‐off values further enhances the practical application of these findings for tailored risk stratification.

Nonetheless, there are several limitations to consider. The cross‐sectional nature of the study restricts the ability to establish causal relationships between anthropometric measures and type 2 diabetes. Additionally, the focus on middle‐aged and elderly participants from southern Iran may fit the applicability of the findings to younger populations or individuals from different ethnic backgrounds. Although indices like WHR and WHtR were measured accurately, the absence of advanced imaging techniques, such as CT or MRI, limits the direct assessment of visceral fat and central obesity. Additionally, reliance on self‐reported data for variables like smoking and physical activity introduces potential reporting bias. Future longitudinal research involving a variety of populations is essential to validate these results and explore the long‐term predictive significance of these indices for diabetes and other metabolic disorders.

## 5 Conclusion

This study emphasizes the strong association of abdominal obesity indices, particularly WHR and WHtR, with T2DM, outperforming BMI in both genders. WHR proved to be a reliable measure, especially among women, highlighting the importance of assessing central fat distribution. While BMI remains widely used, its limitations in reflecting fat distribution reduce its utility for metabolic risk assessment. These findings support the use of WHR and WHtR as practical tools for early diabetes risk identification, underscoring the need for further longitudinal studies to confirm these associations and refine risk‐stratification strategies.

## Author Contributions

**Seyyed Mohammad Hashemi:** conceptualization, investigation, project administration, data curation. **Elaheh Salarpour:** conceptualization, visualization, writing – original draft, formal analysis. **Parsa Saberian:** writing – original draft, writing – review and editing, methodology. **Arezoo Ghazalgoo:** investigation, data curation, writing – original draft. **Sara Parandavar:** conceptualization, data curation, writing – original draft. **Ehsan Amini‐Salehi:** writing – original draft, software, formal analysis. **Mohammad‐Hossein Keivanlou:** writing – original draft, writing – review and editing. **Ladan Hajiabdolrassouli:** supervision, data curation, conceptualization, writing – review and editing, investigation, formal analysis, software.

## Ethics Statement

The research received ethical approval from the Ethics Committee under the code IR.HUMS.REC.1401.295 as a component of the Persian cohort study, funded by Hormozgan University of Medical Sciences. The study adhered strictly to ethical principles, including ensuring originality and obtaining informed consent, which were meticulously overseen by the researchers.

## Consent

Informed consent was obtained from all subjects and/or their legal guardian(s).

## Conflicts of Interest

The authors declare no conflicts of interest.

## Transparency Statement

The lead author Ladan Hajiabdolrassouli affirms that this manuscript is an honest, accurate, and transparent account of the study being reported; that no important aspects of the study have been omitted; and that any discrepancies from the study as planned (and, if relevant, registered) have been explained.

## Artificial Intelligence Assistance Disclosure

Artificial intelligence (ChatGPT by OpenAI) tools were used solely for grammar correction, fluency improvement, and textual consistency. Their role was limited to language refinement and readability enhancements, while the content, ideas, arguments, and conclusions of this article were created and reviewed by all authors. No AI‐generated material was used for research, references, data, graphics, statistics, or methodology, and all analysis and sources were independently developed and verified. All authors have read the manuscript and take responsibility for its content.

## Data Availability

The data that support the findings of this study are available on request from the corresponding author. The data are not publicly available due to privacy or ethical restrictions.
